# Endocrine Late Effects in Survivors of Cancer in Adolescence and Young Adulthood: A Danish Population-Based Cohort Study

**DOI:** 10.1001/jamanetworkopen.2018.0349

**Published:** 2018-06-29

**Authors:** Mette Vestergaard Jensen, Kathrine Rugbjerg, Sofie de Fine Licht, Christoffer Johansen, Kjeld Schmiegelow, Klaus Kaae Andersen, Jeanette Falck Winther

**Affiliations:** 1 Survivorship Unit, Danish Cancer Society Research Center, Copenhagen, Denmark; 2 Department of Clinical Medicine, Juliane Marie Centre, Rigshospitalet, Copenhagen, Denmark; 3 Statistics, Bioinformatics, and Registries, Danish Cancer Society Research Center, Copenhagen, Denmark; 4 Department of Clinical Medicine, Faculty of Health, Aarhus University, Aarhus, Denmark

## Abstract

**Importance:**

As survival rates from cancer have improved dramatically over the last decades, there is a need to explore the long-term consequences. Adolescents and young adults with cancer are at risk for several therapy-related late effects; however, these have not been studied extensively.

**Objective:**

To investigate the lifetime risks of endocrine late effects of cancer and cancer treatment in adolescent and young adult cancer survivors.

**Design, Setting, and Participants:**

This Danish, nationwide, population-based cohort study was conducted from January 1, 1976, through December 31, 2009, and included follow-up from January 1, 1977, through December 31, 2010. A total of 32 548 one-year cancer survivors diagnosed at ages 15 to 39 years were identified using the Danish Cancer Registry and 188 728 cancer-free comparison participants matched by year of birth and sex were randomly chosen from the Danish Civil Registration system. Analyses were performed from July 3, 2015, to February 27, 2018.

**Exposures:**

Individuals in the survivor cohort were diagnosed with a first primary cancer at ages 15 to 39 years and received treatment according to recommendations and guidelines at time of diagnosis.

**Main Outcomes and Measures:**

By linkage to the National Patient Register, all hospital contacts for endocrine diseases were identified, and standardized hospitalization rate ratios (RRs) and absolute excess risks (AERs) were calculated.

**Results:**

A total of 32 548 adolescent and young adult 1-year cancer survivors (14 021 [43.1%] male) in the Danish Patient Registry were followed up for 379 157 person-years (median [range]: 10 [0-34] years) and 188 728 cancer-free participants (82 669 [43.8%] male) for comparison were followed up for 2 958 994 person-years (median [range]: 15 [0-34] years). A total of 2129 survivors (6.5%) had at least 1 hospital contact for an endocrine disease, while 1232.0 (3.8%) were expected, yielding a statistically significant increased RR of 1.73 (95% CI, 1.65-1.81). The RRs were highest for testicular hypofunction (75.12; 95% CI, 45.99-122.70), ovarian hypofunction (14.65; 95% CI, 8.29-25.86), and pituitary hypofunction (11.14; 95% CI, 8.09-15.34). The leading reasons for hospital contacts were thyroid disease (38.0% of total AER), testicular dysfunction (17.1% of total AER), and diabetes (14.4% of total AER). Leukemia survivors were at a high risk for any endocrine disease (RR, 3.97; 95% CI, 3.10-5.09), while Hodgkin lymphoma survivors (RR, 3.06; 95% CI, 2.62-3.57) had the highest disease-specific excess risk for hypothyroidism (AER, 362 per 100 000 person-years; 95% CI, 280-443 per 100 000 person-years).

**Conclusions and Relevance:**

The increased risk for endocrine diseases in adolescent and young adult cancer survivors indicates the need for counseling and follow-up, and could guide future preventive measures and surveillance strategies. Additional studies are required to determine exact associations between treatment regimens and endocrine diseases.

## Key Points

**Question:**

Are adolescent and young adult cancer survivors at increased risk for endocrine diseases?

**Findings:**

This Danish population-based cohort study that included 32 548 adolescent and young adult cancer survivors shows a 73% higher risk for endocrine diseases in these cancer survivors than a matched cancer-free population. This study shows the patterns of endocrine late effects associated with many cancer sites and how they were modified by patient factors.

**Meaning:**

This study represents the first step in identifying patients who are at risk for endocrine late effects and indicates the need for surveillance of these patients to prevent the most severe conditions.

## Teaser

This population-based cohort study uses Danish Cancer Registry data to investigate the lifetime risk of endocrine late effects of cancer and cancer treatment, including pituitary and gonadal hypofunction, in patients diagnosed and treated as adolescents and young adults.

## Introduction

Adolescent and young adult oncology has recently become a subspecialty of cancer research.^1,2^ Adolescent and young adult cancer survivors, defined as those in whom cancer was diagnosed when they were aged 15 to 39 years,^2,3,4^ differ from younger and older patients with cancer in the biology, epidemiology, and clinical outcomes of cancer^5^ and are at risk for long-term morbidity associated with their cancer or cancer treatment. Cancer is 7-fold more frequent in adolescents and young adults than in children younger than 15 years,^3,4^ with the pattern of late effects depending on the age at diagnosis.^6^ Most data on the long-term sequelae in cancer survivors at a young age are derived from studies of childhood cancer survivors.^3,7^ Survivors of cancer in adolescence and young adulthood, their relatives, and the treating clinicians also require information on the long-term outcomes of treatment.

Common late effects in this population of survivors include second primary cancers, cardiovascular and pulmonary complications, neurological complications, and endocrine and gonadal disorders.^6^ These are stages of life with many transitions in terms of education, employment, social relations, relocations, and family formation.^3^ Endocrine late effects, with hormonal disturbances and gonadal dysfunction, could have many physical and social consequences for cancer survivors. Several studies have assessed the late effects of treatment for site-specific cancers in the age range of adolescents and young adults,^3^ including testicular cancer,^8^ Hodgkin lymphoma,^9,10,11^ and breast cancer,^12^ reporting increased risks for hypogonadism, hypothyroidism, and premature menopause in survivors. However, there is little information about endocrine late effects in survivors of other cancers in this age group. We report the results of, to our knowledge, the first large-scale population-based study of all hospital contacts for endocrine diseases, which includes more than 32 000 adolescent and young adult 1-year cancer survivors and 5-fold as many population comparisons.

## Methods

### Survivors and Comparison Participants

The basic cohort of adolescent and young adult cancer survivors was identified from the Danish Cancer Registry,^13^ which contains records of all incident cases of cancer nationwide since 1943. Each cancer record includes the personal identification number, date of diagnosis, and type of cancer. Cancers were classified according to modified *International Classification of Diseases, Seventh Revision *codes between 1943 and 1977 and according to the *International Classification of Diseases, Tenth Revision* (*ICD*-*10*) for diagnosis and the *International Classification of Diseases for Oncology, Third Revision* for morphology and topography from 1978 onward.^13^ The unique personal identification number assigned to all Danish citizens since the start of the Civil Registration System on April 2, 1968, incorporates date of birth and sex and permits accurate linkage among Danish administrative and medical registers.^14,15^ From 1976 through 2009, 38 670 individuals aged 15 to 39 years were registered with a first primary cancer in the Danish Cancer Registry. These individuals were the basis for the 1-year survivor cohort to observe in the Danish National Patient Registry, which is a nationwide registry initiated on January 1, 1977 (eFigure 1 in the Supplement). We followed the Strengthening the Reporting of Observational Studies in Epidemiology (STROBE) reporting guideline. This study was approved by the Danish Data Protection Agency. Patient consent was waived because the data set did not include any identifiable sensitive personal data or identification number.

The comparison cohort was identified through the Civil Registration System.^14,15^ For each survivor, we randomly chose 5 cancer-free comparison participants of the same sex and year of birth who were alive on the date of diagnosis of cancer in the survivor, resulting in 193 350 comparisons. In both cohorts, we excluded individuals who had died or emigrated during the first year after cancer diagnosis or a corresponding date for the comparison participants. This resulted in a cohort of 34 448 survivors and 192 254 comparison participants (eFigure 1 in the Supplement).

### Hospital Contacts for Endocrine Disease

For both survivors and comparison participants, we identified all hospital admissions and outpatient visits with a primary or supplementary discharge diagnosis of endocrine disease (*International Classification of Diseases, Eighth Revision* [*ICD-8*] codes 240-258 and *ICD-10* codes E01-E35 and E89) in the Danish Patient Registry.^16^ The registry includes information on all hospital admissions for somatic disease since 1977, including outpatient visits since 1995. Diagnoses made by general practitioners were not included. Diagnostic information was coded according to *ICD-8* until 1993 and thereafter according to *ICD-10*.^16^ We identified hospital admissions for endocrine diseases defined in chapter 3 of *ICD-8* and chapter 4 of *ICD-10* and excluded nutritional and metabolic diseases, including metabolic syndrome, as we consider these diseases multifactorial and strongly associated with lifestyle or other factors rather than with cancer treatment. Further, metabolic syndrome was introduced in *ICD-10* only recently. We also excluded congenital endocrine disorders, disorders of puberty, and diseases of the thymus, which occur primarily in childhood.

We excluded patients in whom an endocrine disease had been diagnosed before the cancer or a corresponding date for comparison participants as well as survivors with chromosome abnormalities or congenital endocrine malformations (eFigure 1 in the Supplement) registered in the Danish Patient Registry. We also excluded patients with a pituitary tumor because of the direct risk for endocrine dysfunction. After these exclusions, 32 548 one-year survivors and 188 728 population comparison participants remained for analysis.

### Follow-up

Follow-up for endocrine disease late effects started 1 year after cancer diagnosis and on a corresponding date for the equivalent comparison participants. Follow-up ended on the date of death, emigration, second primary cancer diagnosis in survivors or first primary cancer diagnosis in comparison participants, or the closing date (December 31, 2010), whichever occurred first. For participants with more than 1 hospital contact for a particular endocrine disease, only the first record was retained, which was presumed to be the date of diagnosis.

### Statistical Analysis

Risk analyses were performed for any endocrine disease and for 9 main diagnostic categories and 26 subcategories. We compared the rates of first hospital contact for survivors with expected rates derived from the comparison cohort. We estimated hospitalization rate ratios (RRs) with 95% confidence intervals as the ratio of observed to expected hospitalization rates for each defined disease entity separately and by censoring for competing events. We used Fieller theorem, assuming that the observed number of first hospital contacts followed a Poisson distribution.^17^ We calculated absolute excess risks (AERs)—the additional risk of adolescent and young adult cancer survivors for a hospital contact for an endocrine disease—as the difference between the observed and the expected first hospitalization rate for endocrine disease per 100 000 person-years of follow-up, with 95% confidence intervals. Risk analyses were performed for the total cohort of survivors and separately in subcohorts of survivors who had 1 of the 10 most frequent cancers occurring in adolescence and young adulthood. Analyses were stratified according to sex, age at cancer diagnosis, calendar period of cancer diagnosis, and attained age at diagnosis of the endocrine disease. The association of survivor demographic characteristics (sex, age, year of diagnosis, and type of cancer) with the risk for any endocrine disease was investigated in a multivariate analysis within the survivor cohort. A Cox proportional hazards model was used to estimate hospitalization rate hazard ratios for any endocrine disease. All tests were 2-sided likelihood ratio tests with a *P* value less than .05 considered statistically significant. We estimated the cumulative incidence of overall and selected endocrine diseases, with death as a competing risk^18^ and age as the underlying timescale using left truncation. Statistical analyses were conducted using SAS software version 9.3 (SAS Institute Inc) and R statistical computing software version 3.2.3 (R Foundation), and the Survival and Design packages were used.

## Results

We studied 32 548 adolescent and young adult 1-year cancer survivors (14 021 [43.1%] male) in the Danish Patient Registry for 379 157 person-years (median [range] time, 10 [0-34] years) and 188 728 population comparisons (82 669 [43.8%] male) for 2 958 994 person-years (median [range] time, 15 [0-34] years). A total of 2129 survivors (6.5%) had at least 1 hospital contact for an endocrine disease, while 1232.0 (3.8%) were expected, yielding a statistically significantly increased RR of 1.73 (95% CI, 1.65-1.81) (Table 1). The overall observed and expected rates of hospitalization for endocrine diseases were 561.5 and 324.9 per 100 000 person-years, respectively, resulting in an AER of 236.6 (95% CI, 212-261) per 100 000 person-years (ie, a new excess case of endocrine disease in 237 of 100 000 survivors for each additional year of follow-up) (Table 1). The risk for any endocrine disease compared with risk in the background population was higher in male survivors (RR, 2.41; 95% CI, 2.23-2.61) than in female survivors (RR, 1.46; 95% CI, 1.38-1.55), reflecting lower background rates of endocrine diseases in males. A younger age at cancer diagnosis was associated with a higher RR and a higher AER for any endocrine disease (Table 1). The risk for hospital contact for endocrine diseases decreased with time since cancer diagnosis, with an RR of 2.60 (95% CI, 2.39-2.82) for 1 to 4 years since diagnosis and an RR of 1.45 (95% CI, 1.34-1.57) for 10 to 19 years since diagnosis (Table 1).

**Table 1.** Numbers of First Hospital Contacts for Endocrine Disease by Patient Factors

Characteristic	Hospital Contacts, No.	Survivors at Risk, Person-Years	All Endocrine Disease					
			First Hospital Contacts, No.^a^			Hospitalization Rate per 100 000 Person-Years		
			Observed	Expected	RR (95% CI)	Observed	Expected	AER (95% CI)
Total^b^	32 548	379 157	2129	1232.0	1.73 (1.65 to 1.81)	561.5	324.9	237 (212 to 261)
Sex								
Male	14 021	167 616	826	342.5	2.41 (2.23 to 2.61)	492.8	204.3	288 (254 to 323)
Female	18 527	211 541	1303	889.5	1.46 (1.38 to 1.55)	616.0	420.5	195 (160 to 230)
Attained age, y								
16-19	1369	2128	17	2.0	8.43 (4.14 to 17.16)	798.8	94.8	704 (321 to 1087)
20-29	9363	34 812	197	49.9	3.95 (3.31 to 4.70)	565.9	143.4	423 (342 to 503)
30-39	26 930	121 540	603	261.3	2.31 (2.10 to 2.53)	496.1	215.0	281 (240 to 322)
40-49	21 463	145 634	728	470.5	1.55 (1.43 to 1.68)	499.9	323.1	177 (139 to 215)
50-59	9997	60 499	410	331.3	1.24 (1.12 to 1.37)	677.7	547.6	130 (62 to 199)
60-69	3539	14 152	166	113.5	1.46 (1.24 to 1.72)	1172.9	802.1	371 (187 to 555)
≥70	285	391	8	3.5	2.29 (1.08 to 4.87)	2044.2	890.9	1153 (−287 to 2594)
Age at cancer diagnosis, y								
15-19	1838	21 014	139	34.4	4.04 (3.41 to 4.78)	661.5	163.8	498 (387 to 608)
20-24	3328	42 100	248	88.7	2.80 (2.46 to 3.17)	589.1	210.7	378 (305 to 452)
25-29	5920	73 297	362	200.0	1.81 (1.63 to 2.01)	493.9	272.9	221 (170 to 272)
30-34	8939	104 625	568	349.1	1.63 (1.50 to 1.77)	542.9	333.7	209 (164 to 254)
35-39	12 523	138 121	812	559.9	1.45 (1.35 to 1.56)	587.9	405.3	183 (142 to 224)
Calender year for cancer diagnosis								
1975-1989	12 411	217 562	1036	717.9	1.44 (1.35 to 1.54)	476.2	330.0	146 (116 to 176)
1990-2009	20 137	161 595	1093	514.2	2.13 (2.00 to 2.26)	676.4	318.2	358 (318 to 399)
Years since cancer diagnosis								
1-4	32 548	109 682	616	237.1	2.60 (2.39 to 2.82)	561.6	216.1	345 (301 to 390)
5-9	23 874	100 059	421	254.0	1.66 (1.50 to 1.83)	420.8	253.9	167 (126 to 208)
10-19	17 642	121 623	649	448.8	1.45 (1.34 to 1.57)	533.6	369.0	165 (123 to 206)
>20	8325	47 793	443	292.2	1.52 (1.38 to 1.67)	926.9	611.3	315 (229 to 402)

Abbreviations: AER, absolute excess risk; RR, hospitalization rate ratio.

^a^ Observed and expected numbers of first hospital contacts for endocrine disease of any type among 32 548 adolescent and young adult 1-year cancer survivors in Denmark, 1976 to 2009.

^b^ According to Statistics Denmark, 8% of the Danish population has immigrated from non-Western countries.

Survivors had significantly increased risks for 8 of 9 main diagnostic groups of endocrine disease and 18 of 26 subcategories (Table 2). The highest RRs were seen for testicular hypofunction (75.12; 95% CI, 45.99-122.70), ovarian hypofunction (14.65; 95% CI, 8.29-25.86), and pituitary hypofunction (11.14; 95% CI, 8.09-15.34). Diseases of the thyroid gland, testicular dysfunction, and diabetes were the leading reasons for hospital contacts (38.0%, 17.1%, and 14.4% of total AER, respectively).

**Table 2.** First Hospital Contacts for Endocrine Disease by Diagnostic Categories or Diagnoses^a^

Category of Endocrine Disease and Diagnostic Entity (*ICD-10 *Code)^b^	First Hospital Contacts, No.^c^		RR (95% CI)	AER (95% CI)	% of Total AER
	Observed	Expected			
Diseases of the thyroid gland (E01, E02, E03.2-E03.9, E04-E07, E35.0, and E89.0)	1039	654.9	1.59 (1.48 to 1.70)	100 (83 to 117)	38.0
Goiter, nontoxic (E01 and E04)	394	325.6	1.21 (1.09 to 1.35)	18 (7 to 28)	
Thyrotoxicosis (E05)	279	225.0	1.24 (1.09 to 1.41)	14 (5 to 23)	
Hypothyroidism (E02, E03.2-E03.9, and E89.0)	444	152.4	2.91 (2.61 to 3.25)	75 (64 to 85)	
Thyroiditis (E06)	55	46.8	1.17 (0.88 to 1.56)	2 (−2 to 6)	
Other diseases of the thyroid gland (E07 and E35.0)	14	15.5	0.91 (0.52 to 1.58)	0 (−2 to 2)	
Diabetes (E10-E14 and E89.1)	660	511.1	1.29 (1.19 to 1.40)	38 (25 to 52)	14.4
Type 1 (E10)	264	188.4	1.40 (1.23 to 1.60)	19 (11 to 28)	
Type 2 (E11)	520	408.4	1.27 (1.16 to 1.40)	29 (16 to 41)	
Other types (E12-E14 and E89.1)	146	89.4	1.63 (1.37 to 1.95)	14 (8 to 21)	
Disorders of pancreatic internal secretion other than diabetes (E15-E16)	49	41.1	1.19 (0.88 to 1.61)	2 (−2 to 6)	0.8
Diseases of parathyroid glands (E20-E21, and E89.2)	93	33.8	2.75 (2.17 to 3.49)	15 (10 to 20)	5.7
Hyperparathyroidism (E21.0-E21.3)	48	26.4	1.82 (1.33 to 2.49)	6 (2 to 9)	
Hypoparathyroidism (E20-E89.2)	49	7.9	6.19 (4.24 to 9.04)	10 (7 to 14)	
Other diseases of parathyroid glands (E21.4 and E21.5)	1	0.4	2.53 (0.26 to 24.42)	0 (0 to 1)	
Diseases of pituitary gland (E22-E23 and E89.3)	122	22.1	5.53 (4.38 to 6.99)	25 (20 to 31)	9.5
Pituitary hyperfunction (E22)	19	12.0	1.58 (0.96 to 2.59)	2 (0 to 4)	
Pituitary hypofunction (E23.0-E23.3 and E89.3)	93	8.4	11.14 (8.09 to 15.34)	22 (17 to 26)	
Other diseases of pituitary gland (E23.6 and E23.7)	23	2.8	8.28 (4.58 to 14.96)	5 (3 to 8)	
Diseases of adrenal glands (E25.8, E25.9, E26, E27, E35.1, and E89.6)	73	18.25	4.00 (3.01 to 5.31)	14 (10 to 18)	5.3
Adrenocortical hyperfunction (E25.8, E25.9, E26, and E27.0)	12	5.5	2.17 (1.14 to 4.13)	2 (0 to 3)	
Adrenomedullary hyperfunction (E27.5)	14	1.6	8.66 (4.02 to 18.68)	3 (1 to 5)	
Adrenal hypofunction (E27.1-E27.4 and E89.6)	43	7.7	5.58 (3.76 to 8.28)	9 (6 to 12)	
Other diseases of adrenal glands (E27.8, E27.9, and E35.1)	6	4.1	1.46 (0.61 to 3.51)	0 (−1 to 2)	
Ovarian dysfunction (E28 and E89.4)^d^	74	35.4	2.09 (1.61 to 2.70)	10 (5 to 14)	3.8
Ovarian hypofunction (E28.3 and E89.4)	35	2.4	14.65 (8.29 to 25.86)	8 (5 to 11)	
Other diseases of the ovaries (E28.2, E28.8, and E28.9)	39	31.5	1.24 (0.88 to 1.74)	2 (−1 to 5)	
Testicular dysfunction (E29 and E89.5)^d^	182	4.5	40.24 (27.91 to 58.01)	45 (38 to 52)	17.1
Testicular hypofunction (E29.1 and E89.5)	175	2.3	75.12 (45.99 to 122.70)	44 (37 to 51)	
Other diseases of testis (E29.8 and E29.9)	10	2.4	4.22 (1.95 to 9.16)	2 (0 to 4)	
Other diseases of endocrine glands (E24, E31, E34, E35.8, E89.8, and E89.9)	70	14.7	4.78 (3.54 to 6.45)	14 (10 to 18)	5.3
Cushing syndrome (E24)	10	4.4	2.29 (1.13 to 4.66)	1 (0 to 3)	
Other endocrine diseases (E34, E35.8, E89.8, and E89.9)	59	9.9	5.94 (4.22 to 8.36)	12 (9 to 16)	

Abbreviations: AER, absolute excess risk; *ICD-10*, *International Classification of Diseases, Tenth revision*; RR, hospitalization rate ratio.

^a^ Excluded diagnoses are nutritional and metabolic diseases (E40-E88 and E90), metabolic syndrome (E888C), congenital endocrine disorders (E00, E03.0, E03.1, and E25), disorders of puberty (E30), and diseases of thymus (E32).

^b^ Endocrine diseases of which there were fewer than 5 cases are not included even if the RR was high and/or statistically significant.

^c^ Observed and expected numbers of first hospital contacts for endocrine disease among 32 548 adolescent and young adult 1-year cancer survivors in Denmark, by 9 main diagnostic categories and 26 subcategories or diagnoses.

^d^ Numbers and rates for ovarian and testicular diseases are calculated for the entire cancer survivor and comparison cohorts even though these disorders occur only in women and men, respectively.

Analyses by sites of cancer revealed the highest RRs for any endocrine disease for survivors of leukemia (RR, 3.97; 95% CI, 3.10-5.09), Hodgkin lymphoma (RR, 3.06; 95% CI, 2.62-3.57), and brain cancer (RR, 3.03; 95% CI, 2.53-3.64) (Table 3; eFigure 2 in the Supplement). Survivors of Hodgkin lymphoma had a particularly high excess risk for hypothyroidism (AER, 362 per 100 000 person-years; 95% CI, 280-443 per 100 000 person-years). Brain cancer survivors were at increased risk for a broad spectrum of endocrine diseases, comprising diseases of the pituitary and thyroid glands as well as diabetes. For survivors of the most common cancers among adolescents and young adults, the risks for hospital contact for endocrine disease were significantly increased for testicular cancer (RR, 2.50; 95% CI, 2.25-2.78) and breast cancer (RR, 1.16; 95% CI, 1.02-1.32), whereas the risks among survivors of malignant melanoma (RR, 1.08; 95% CI, 0.95-1.24) and cervical cancer (RR, 0.94; 95% CI, 0.82-1.07) were similar to those of the comparison cohort (Table 3).

**Table 3.** Endocrine Disease by 10 Most Frequent Cancer Sites^a^

Site of Cancer and Endocrine Disease^b^	Hospital Contacts, No.	RR (95% CI)	AER (95% CI) per 100 000 Person-Years
Brain cancer (n = 1895)	116	3.03 (2.53 to 3.64)	490 (357 to 623)
Pituitary hypofunction	40	112.01 (75.55 to 166.07)	244 (168 to 320)
Other pituitary diseases	15	104.23 (55.19 to 196.85)	90 (44 to 137)
Pituitary hyperfunction	5	11.28 (4.57 to 27.84)	28 (1 to 54)
Thyrotoxicosis	14	2.24 (1.32 to 3.79)	47 (2 to 92)
Diabetes (all types)	32	2.05 (1.44 to 2.90)	100 (32 to 168)
Leukemia (n = 944)	63	3.97 (3.10 to 5.09)	755 (506 to 1004)
Ovarian hypofunction	13	236.74 (121.17 to 462.56)	200 (91 to 309)
Testicular hypofunction	8	155.26 (69.59 to 346.42)	122 (37 to 207)
Pituitary hypofunction	8	52.56 (25.28 to 109.27)	121 (35 to 206)
Type 2 diabetes	17	3.46 (2.14 to 5.57)	187 (62 to 313)
Hodgkin lymphoma (n = 1713)	165	3.06 (2.62 to 3.57)	509 (393 to 624)
Testicular hypofunction	5	27.67 (10.53 to 72.72)	21 (2 to 40)
Hypothyroidism	87	14.89 (11.93 to 18.59)	362 (280 to 443)
Diabetes, other and unspecified	11	2.68 (1.48 to 4.87)	30 (2 to 58)
Goiter	32	2.46 (1.73 to 3.49)	83 (35 to 132)
Type 2 diabetes	37	2.00 (1.45 to 2.77)	81 (29 to 134)
Non-Hodgkin lymphoma (n = 1201)	66	1.86 (1.46 to 2.37)	237 (113 to 361)
Ovarian hypofunction	9	134.34 (58.76 to 307.11)	67 (23 to 112)
Testicular hypofunction	6	57.88 (23.58 to 142.07)	44 (8 to 81)
Pituitary hypofunction	5	17.89 (7.19 to 44.48)	36 (3 to 69)
Hypothyroidism	14	3.78 (2.23 to 6.42)	78 (22 to 134)
Testis (n = 5503)	393	2.50 (2.25 to 2.78)	305 (254 to 356)
Testicular hypofunction	148	134.71 (82.75 to 219.29)	186 (156 to 216)
Adrenal hypofunction	21	21.31 (11.14 to 40.77)	25 (14 to 37)
Testicular dysfunction, other and unspecified	8	7.23 (3.14 to 16.60)	9 (2 to 16)
Thyrotoxicosis	24	2.21 (1.45 to 3.39)	17 (4 to 29)
Type 1 diabetes	73	1.58 (1.24 to 2.00)	34 (12 to 55)
Type 2 diabetes	143	1.50 (1.27 to 1.78)	61 (30 to 91)
Ovary (n = 775)	44	1.14 (0.85 to 1.54)	56 (−78 to 189)
Diabetes, other and unspecified	7	4.18 (1.98 to 8.86)	53 (1 to 104)
Breast (n = 4654)	237	1.16 (1.02 to 1.32)	71 (7 to 136)
Diseases of the thyroid gland	172	1.28 (1.10 to 1.49)	80 (26 to 135)
Malignant melanoma (n = 5133)	225	1.08 (0.95 to 1.24)	30 (−21 to 82)
Goiter	82	1.44 (1.16 to 1.80)	43 (13 to 73)
Colon (n = 596)	14	0.68 (0.40 to 1.14)	−111 (−232 to 11)
Cervix (n = 3987)	224	0.94 (0.82 to 1.07)	−26 (−80 to 28)
Type 2 diabetes	73	1.31 (1.03 to 1.65)	31 (0 to 61)

Abbreviations: AER, absolute excess risk, RR, hospitalization rate ratio.

^a^ Only endocrine diseases for which the RR had a lower 95% confidence limit of 1 or greater and AERs with a lower 95% confidence limit of 0 or greater were included. Endocrine disease categories of which there were fewer than 5 cases, however, were not included, even if the RR was high and/or statistically significant.

^b^ Sample size indicate number of survivors.

When the survivors were stratified by period of treatment, the RR of hospital contact for endocrine disease for those diagnosed during the period of 1975 to 1989 was 1.44 (95% CI, 1.35-1.54), whereas the corresponding risk for those diagnosed during the period of 1990 to 2009 was 48% higher (RR, 2.13; 95% CI, 2.00-2.26) (Table 1).

Overall, RRs and AERs diminished with age (Table 1 and Figure). The risk for diabetes increased markedly after age 50 years for both survivors and comparison participants, while the RR and AER for hypothyroidism were highest in the age group 20 to 39 years. The vast majority of cases of pituitary hypofunction were diagnosed before the age of 30 years.

**Figure.**
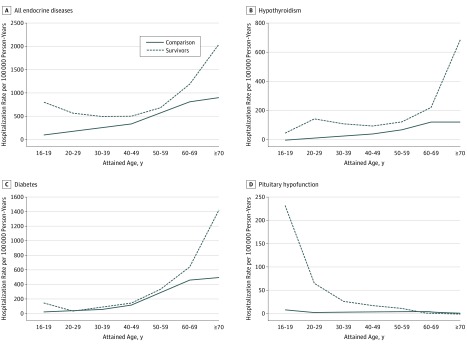
Hospitalization Rates for Any Endocrine Disease and Selected Endocrine Diseases Age-specific observed and expected hospitalization rates for any endocrine disease and selected endocrine diseases among 32 548 adolescent and young adult 1-year cancer survivors.

The cumulative risk of survivors for any endocrine disease by age 30 years was 6.6% (95% CI, 6.5%-6.6%) and by age 60 years was 12.5% (95% CI, 12.2%-12.7%), while the cumulative risk of comparison participants by age 30 years was 1.7% (95% CI, 1.7%-1.7%) and by age 60 years was 11.4% (95% CI, 11.2%-11.6%). Survivors of Hodgkin lymphoma were at considerably high risk for hypothyroidism, with a cumulative risk of 9.6% (95% CI, 7.5%-11.6%) by age 60 years; the corresponding cumulative risk for comparisons was 1.4% (95% CI, 1.3%-1.4%).

In a multivariate analysis, significant variation in the risk of the survivor cohort for any endocrine disorder was found by sex, age at diagnosis, year of diagnosis, and type of cancer (Table 4). The adjusted hazard ratio of females for any endocrine disorder was 1.85 (95% CI, 1.65-2.09; *P* < .001) compared with males. In line with our findings in Table 1, the adjusted hazard ratios decreased significantly with increasing age at cancer diagnosis (ages 35-39 vs 15-19 years: adjusted HR, 0.55; 95% CI, 0.43-0.71; *P* < .001) and increased in the most recent calendar period of diagnosis (1990-2009 vs 1975-1989: adjusted HR, 1.97; 95% CI, 1.78-2.18; *P* < .001). The adjusted hazard ratios according to cancer site matched the corresponding RR estimates compared with the general population (Table 3 and Table 4; eFigure 2 in the Supplement); ie, in both analyses, survivors of leukemia and Hodgkin lymphoma had higher risks for endocrine disease than survivors of brain cancer, whereas survivors of cancers at other sites had lower risks.

**Table 4.** Adjusted Hospitalization Rate Ratios for Endocrine Disease in a Within-Survivor Cohort Analysis

Characteristic	First Hospitalizations, No.	Adjusted HR (95% CI)	*P* Value
Sex			<.001
Male	826	1 [Reference]	NA
Female	1303	1.85 (1.65-2.09)	<.001
Age at cancer diagnosis, y			<.001
15-19	139	1 [Reference]	NA
20-24	248	0.89 (0.70-1.12)	.32
25-29	362	0.67 (0.53-0.85)	.001
30-34	568	0.63 (0.50-0.81)	<.001
35-39	812	0.55 (0.43-0.71)	<.001
Calendar period of cancer diagnosis, y			<.001
1975-1989	1036	1 [Reference]	NA
1990-2009	1093	1.97 (1.78-2.18)	<.001
Site of cancer^a^			<.001
Leukemia (n = 944)	63	1.36 (1.00-1.85)	.05
Hodgkin lymphoma (n = 1713)	165	1.05 (0.83-1.33)	.70
Brain cancer (n = 1895)	116	1 [Reference]	NA
Testicular cancer (n = 5503)	393	0.92 (0.74-1.15)	.45
Non-Hodgkin lymphoma (n = 1201)	66	0.69 (0.51-0.93)	.02
Breast cancer (n = 4654)	237	0.48 (0.38-0.61)	<.001
Ovarian cancer (n = 775)	44	0.45 (0.32-0.64)	<.001
Malignant melanoma (n = 5133)	225	0.43 (0.34-0.54)	<.001
Cervical cancer (n = 3987)	224	0.39 (0.31-0.49)	<.001
Colon cancer (n = 596)	14	0.28 (0.16-0.49)	<.001

Abbreviations: HR, hazard ratio; NA, not applicable.

^a^ Sample sizes indicate number of survivors.

## Discussion

This nationwide, population-based cohort study showed that adolescent and young adult cancer survivors were at 73% higher risk for endocrine disease than the background population. The dominating endocrine diseases were thyroid diseases, testicular dysfunction, and diabetes. A particularly high AER for hypothyroidism was observed in Hodgkin lymphoma survivors. Treatment for Hodgkin lymphoma includes irradiation to the thyroid region; previous reviews concluded that half of Hodgkin lymphoma survivors who were irradiated subsequently experienced thyroid disease,^10,19^ and the reported risk increased with radiation dose. Alkylating chemotherapy and irradiation increased the risk for testicular dysfunction in a dose-dependent manner.^10,19^ In this study, brain cancer survivors had a broad spectrum of endocrine diseases, some of which might be secondary to hypothalamic and pituitary dysfunction. We found particularly high RRs and AERs for pituitary diseases in brain cancer survivors based on 40 observations of hypopituitarism, 5 observations of hyperpituitarism, and 15 observations of other pituitary diseases in 1895 brain cancer survivors. These numbers were lower than those in a British follow-up study of 56 adult nonpituitary brain cancer survivors who received radiation therapy at ages 21 to 45 years.^20^ Hypopituitarism was reported in 41% of survivors, and the risk was associated with radiation dose but not with age at radiation, sex, or chemotherapy.^20^

In this study, the risks for endocrine diseases were highest in leukemia survivors. In a study by Tauchmanovà et al,^21^ in preparation for bone marrow transplantation, some patients with leukemia underwent total body irradiation, which resulted in exceptionally high risks for gonadal dysfunction (95% of women and 47% of men), thyroid dysfunction (46%), and adrenal abnormalities (10%).

We found a 40-fold increased risk for testicular dysfunction. Testicular damage is common in men receiving chemotherapy, and men receiving cytotoxic chemotherapy had a significantly lower testosterone level than healthy controls in a study of men with cancer diagnosed at a mean age of 28.6 years.^22^ Older age at cancer diagnosis and treatment was negatively associated with testosterone level, and testosterone levels were negatively correlated with fasting glucose, insulin, and body fat mass. As an example of adverse health effects of untreated endocrine diseases, we found increased risk of diabetes in testicular cancer survivors, ovarian cancer survivors, and cervical cancer survivors, which might be related to sex hormone deficiencies. Associations between hypogonadism and men with type 2 diabetes are well described, and the relationship is bidirectional.^23^ Type 1 diabetes may be caused by direct damage to the pancreas. A questionnaire-based survey of late effects in cervical cancer survivors showed significantly increased risk for diabetes in women treated with radiotherapy but not in those treated by surgery or by chemotherapy and surgery.^24^

Of testicular cancer survivors, breast cancer survivors, malignant melanoma survivors, and cervical cancer survivors, which are the most common cancers in the cohort, only testicular cancer survivors and breast cancer survivors had significantly increased risk for hospital contact for endocrine disease. To our knowledge, no previous studies have evaluated the risk of endocrine diseases specifically for survivors of these cancers diagnosed in adolescence and young adulthood.

The within-cohort analysis showed that sex modified the risk for endocrine diseases, with female survivors having the highest risks. The difference in the RRs of male and female survivors might reflect the sex differences seen in the general population or biological differences between men and women (eg, female patients appeared to be more vulnerable to adverse effects of cancer treatment than males, perhaps as a result of differences in oxidative stress and body composition).^25^

The patients with the most recent diagnoses of cancer (1990-2009) had a significantly higher risk for endocrine diseases than those with diagnoses before 1990. Surveillance bias might be more likely in the latest period, but the higher risk is more likely to be because of changes in treatment protocols and survival. Survival rates have improved dramatically over many years, resulting in increased risks for late effects.

The strengths of this study include high statistical power because of the very large cohort of cancer survivors. We used nationwide health registries of high-quality dating several decades back, allowing long follow-up and virtually no loss to follow-up. To our knowledge, this is the first large-scale study of the risks for endocrine diseases after cancer in adolescence and young adulthood, with comparisons of risks for a broad range of well-defined endocrine diseases with those expected for a large, well-defined, population-based comparison cohort. Furthermore, we were able to evaluate patterns in endocrine late effects across the spectrum of cancer sites and other patient-related factors. The inclusion of all 1-year adolescent and young adult cancer survivors diagnosed since 1976 minimizes possible selection bias. Only medically verified diagnoses of endocrine diseases were included, which ensures more correct estimates than those in studies based on self-reported data.

### Limitations

Our study had limitations. The limitations include lack of information of less severe conditions diagnosed and treated by general practitioners. Outpatient visits were included in the patient register in 1995, meaning that only the more severe cases that required hospitalization were included before that date. This results in underestimation of the number of cases. Our results might be influenced by surveillance bias, as cancer survivors are observed more closely in the health care system than the general population. This might have caused an overestimation of the risk estimates reported in this study. The Danish Cancer Registry has limited information on cancer stage and treatment, so associations between treatment factors and endocrine late effects cannot be conclusively determined.

## Conclusions

Although increased risks for a wide range of cardiovascular diseases,^26,27^ secondary malignancies,^28,29^ infectious diseases, and digestive diseases^29,30^ have been reported in cancer survivors, late effects in adolescent and young adult cancer survivors have received little attention. Our study provides new, accurate, and detailed information about these survivors and important clinical information on how the risks for such late effects are modified by patient factors. This is the first step in identifying patients who are at risk for endocrine late effects so that individual profiles can be drawn up to assess the probable risk for endocrine disease. This will require prospective capture and close phenotyping of these diseases. We hope that this study will inspire investigators in future studies to determine exact associations between treatment regimens and endocrine disease and ultimately incorporate them into individual, customized treatment plans. Each future adolescent and young adult cancer patient should be offered the least deleterious treatment to ensure high quality of life after cancer while maintaining the good cure rates. Cure has become an insufficient goal.

## Notes

Supplement. **eFigure 1.** Flowchart of the Adolescent and Young Adult Cancer Survivor Cohort With a First Primary Cancer Diagnosed From 1976 to 2009 At Ages 15-39 Years and of Population Comparisons **eFigure 2.** Rate Ratios for Hospital Contacts for Any Endocrine Disorder in Survivors of Cancer At the 10 Most Frequent Cancer Sites in Adolescents and Young Adults
