# Error in Text

**DOI:** 10.1001/jamanetworkopen.2018.2037

**Published:** 2018-07-27

In the Original Investigation titled “Endocrine Late Effects in Survivors of Cancer in Adolescence and Young Adulthood: A Danish Population-Based Cohort Study,”^1^ published June 29, 2018, there was an error in the wording of the last sentence of the first paragraph in the Introduction section. The sentence should have read: “Survivors of cancer in adolescence and young adulthood, their relatives, and the treating clinicians also require information on the long-term outcomes of treatment.” This article was corrected online.
