# Supplementary material for: Endocrine Late Effects in Survivors of Cancer in Adolescence and Young Adulthood: A Danish Population-Based Cohort Study
Source: JAMA Netw Open. 2018 Jun 29;1(2):e180349. doi: 10.1001/jamanetworkopen.2018.0349 (PMC6324403; doi:10.1001/jamanetworkopen.2018.0349)
Supplement: Supplement. — eFigure 1. Flowchart of the Adolescent and Young Adult Cancer Survivor Cohort With a First Primary Cancer Diagnosed From 1976 to 2009 At Ages 15-39 Years and of Population Comparisons eFigure 2. Rate Ratios for Hospital Contacts for Any Endocrine Disorder in Survivors of Cancer At the 10 Most Frequent Cancer Sites in Adolescents and Young Adults [file jamanetwopen-1-e180349-s001.pdf]

Supplementary Online Content

Jensen MV, Rugbjerg K, de Fine Licht S, et al. Endocrine late effects in survivors of cancer in adolescence and young adulthood: a Danish population-based cohort study. *JAMA Netw Open*. 2018;1(2):e180349. doi:10.1001/jamanetworkopen.2018.0349

eFigure 1. Flowchart of the Adolescent and Young Adult Cancer Survivor Cohort With a First Primary Cancer Diagnosed From 1976 to 2009 At Ages 15-39 Years and of Population Comparisons.
eFigure 2. Rate Ratios for Hospital Contacts for Any Endocrine Disorder in Survivors of Cancer At the 10 Most Frequent Cancer Sites in Adolescents and Young Adults

This supplementary material has been provided by the authors to give readers additional information about their work.

© 2018 Jensen MV et al. *JAMA Network Open*.

eFigure 1. Flowchart of the Adolescent and Young Adult Cancer Survivor Cohort With a First Primary Cancer Diagnosed From 1976 to 2009 At Ages 15-39 Years and of Population Comparisons.

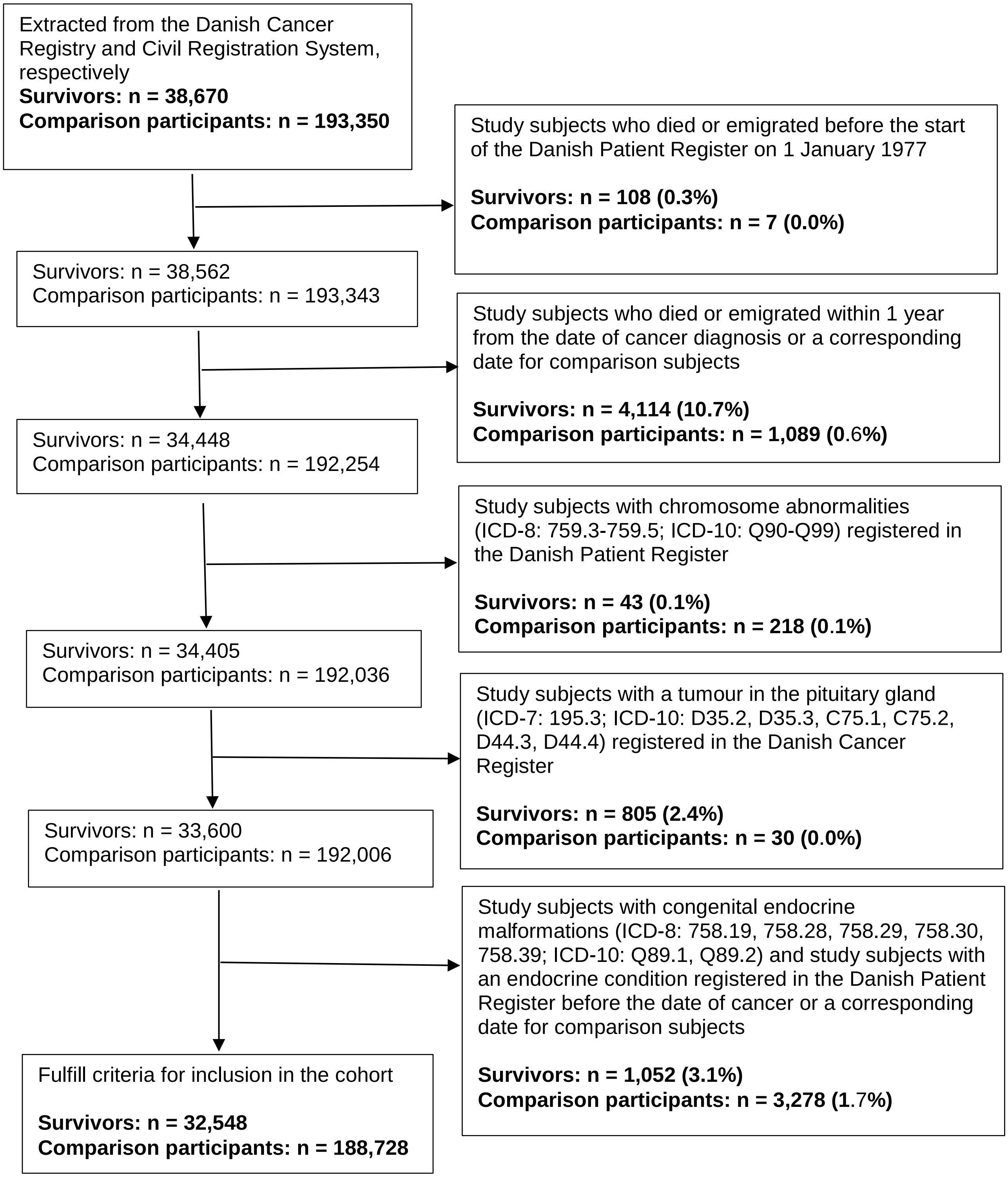

© 2018 Jensen MV et al. *JAMA Network Open*.

Abbreviations: ICD-8, International Classification of Diseases, 8th revision; ICD-10, International Classification of Diseases, 10th revision; ICD-7, International Classification of Diseases, 7th revision.

© 2018 Jensen MV et al. *JAMA Network Open*.

eFigure 2. Rate Ratios for Hospital Contacts for Any Endocrine Disorder in Survivors of Cancer At the 10 Most Frequent Cancer Sites in Adolescents and Young Adults

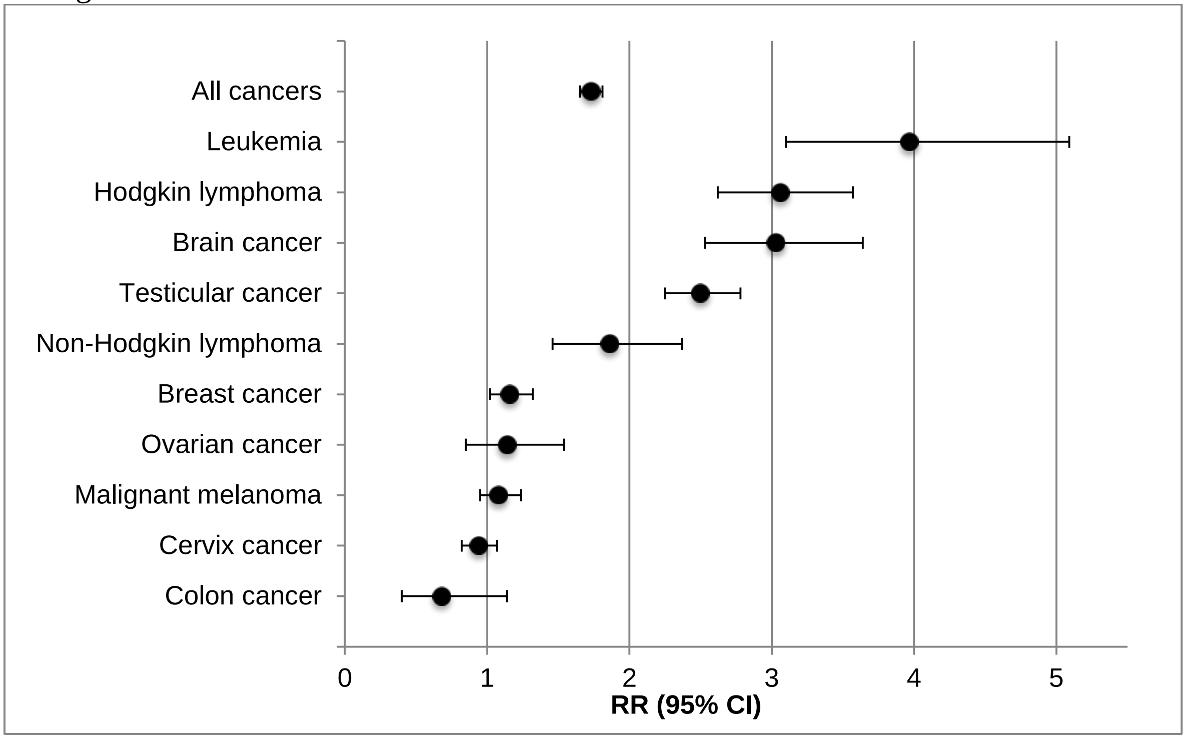

Abbreviations: RR, rate ratios (standardized hospitalization rate ratio); CI, confidence interval.

© 2018 Jensen MV et al. *JAMA Network Open*.
